# [Corrigendum] Influence of aspirin on the CX3CL1/CX3CR1 signaling pathway in acute pulmonary embolism

**DOI:** 10.3892/ijmm.2026.5819

**Published:** 2026-03-31

**Authors:** Zhirong Zhang, Weiji Yang, Rongbiao Ying, Ying Shi, Huifang Jiang, Danli Cai, Jing Kuang, Ruhui Yang, Lingcong Wang

Int J Mol Med 39: 1580-1588, 2017; DOI: 10.3892/ijmm.2017.2969

Following the publication of the above article, an interested reader drew to the authors' attention that, concerning the images showing the construction of the CX3CL1-overexpression adenovirus and the CX3CL1 short hairpin RNA (shRNA) adenovirus in Fig. 1 on p. 1581, the 'P3x100, Ad-CX3CL1 OE' data panel in Fig. 1A contained an overlapping section with the 'P3x100, Ad-CX3CL1 shRNA1' data panel in Fig. 1B, such that these were apparently derived from the same original source where different experiment conditions were reported. In addition, for the lung pathology images shown in Fig. 3 on p. 1584, the images showing the 'ASPx100' and the 'M+Ax100' experiments were apparently identical, suggesting that this figure had also been assembled incorrectly.

After re-examining their original data, the authors regret that Figs. 1 and 3 did contain errors in terms of their assembly, as identified by the external reader. Concerning Fig. 1, the authors wish to point out that these experiments were performed by the virus packaging company (Hanbio Biotechnology), who acknowledged that they were responsible for the error that was made in the provision of the images for this figure. Moreover, this error did not have any real significance in terms of the reported reports in this study, since neither shRNA1 nor shRNA2 was ultimately selected as the vector for the subsequent experiments; shRNA3 was selected as the interference vector of choice. Therefore, the Editor has approved the inclusion of a new version of Fig. 1 in this Corrigendum, comprising only the data for shRNA3 in Fig. 1B. Regarding Fig. 3, the revised version of this is also shown in the subsequent pages, showing the correct data for the 'Nx100', 'ASPx100' and 'M+Ax100' panels.

Note that the revisions made to these figures do not affect the overall conclusions reported in the paper. The authors express their gratitude to the Editor of *International Journal of Molecular Medicine* for allowing them the opportunity to publish this corrigendum, and apologize to the readership for any inconvenience caused.

## Figures and Tables

**Figure 1:**
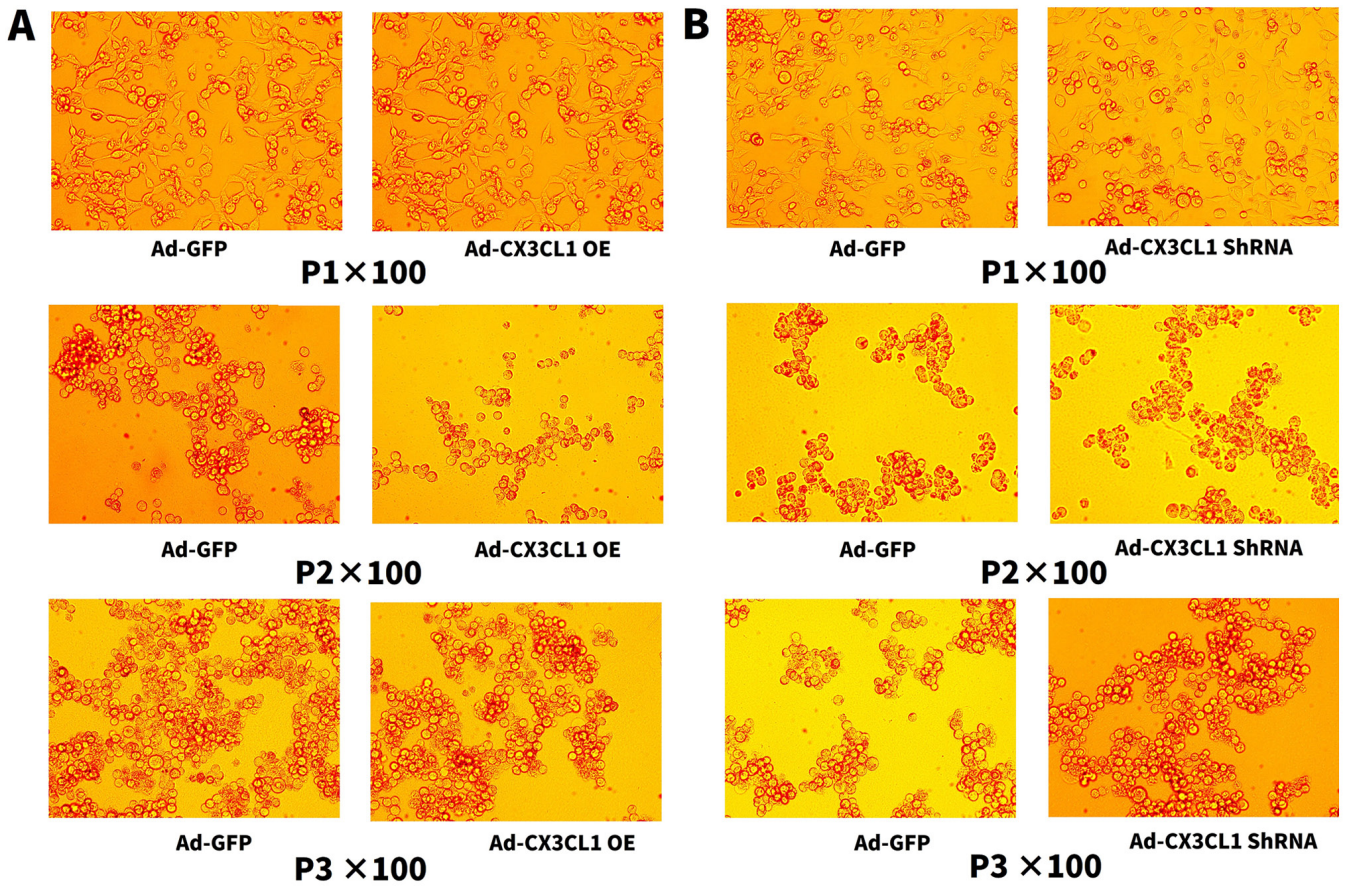
(A) Construction of the CX3CL1-overexpression adenovirus. (B) CX3CL1 short hairpin RNA (shRNA) adenovirus construction.

**Figure 3:**
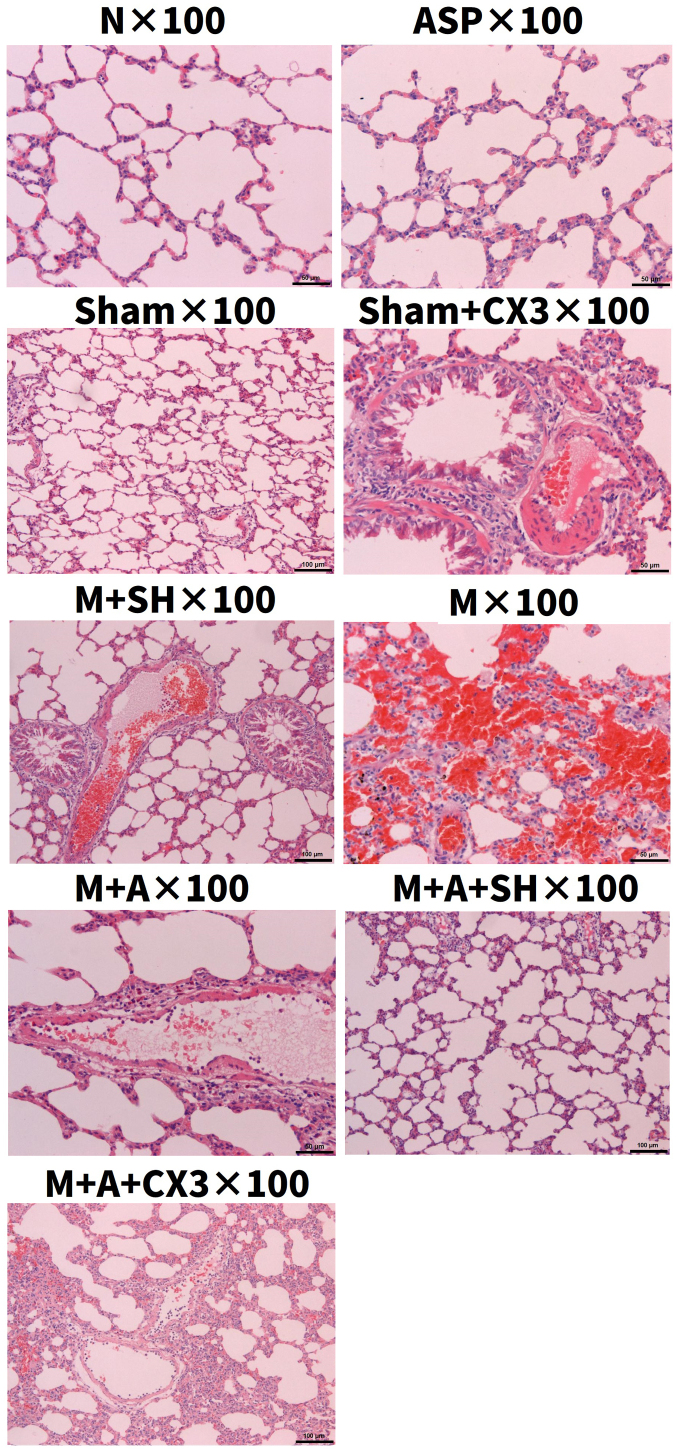
Lung pathology (magnification, x100).

